# Autophagy and Renal Fibrosis

**DOI:** 10.14336/AD.2021.1027

**Published:** 2022-06-01

**Authors:** Shan Liang, Yun-Shan Wu, Dong-Yi Li, Ji-Xin Tang, Hua-Feng Liu

**Affiliations:** ^1^Key Laboratory of Prevention and Management of Chronic Kidney Disease of Zhanjiang, Institute of Nephrology, Affiliated Hospital of Guangdong Medical University, Zhanjiang, Guangdong, China.; ^2^Shunde Women and Children's Hospital, Guangdong Medical University (Foshan Shunde Maternal and Child Healthcare Hospital), Foshan, Guangdong, China.

**Keywords:** autophagy, renal fibrosis, chronic kidney disease, cellular senescence, senescence-associated secretory phenotype

## Abstract

Renal fibrosis is a common process of almost all the chronic kidney diseases progressing to end-stage kidney disease. As a highly conserved lysosomal protein degradation pathway, autophagy is responsible for degrading protein aggregates, damaged organelles, or invading pathogens to maintain intracellular homeostasis. Growing evidence reveals that autophagy is involved in the progression of renal fibrosis, both in the tubulointerstitial compartment and in the glomeruli. Nevertheless, the specific role of autophagy in renal fibrosis has still not been fully understood. Therefore, in this review we will describe the characteristics of autophagy and summarize the recent advances in understanding the functions of autophagy in renal fibrosis. Moreover, the problem existing in this field and the possibility of autophagy as the potential therapeutic target for renal fibrosis have also been discussed.

## 1.Introduction

Renal fibrosis is the common process of chronic kidney disease (CKD) progressing to end-stage renal disease (ESRD) and the mainly histopathologic manifestation of CKD. The global incidence of CKD is about 10% [1], and the incidence of CKD is increasing year by year, becoming one of the major diseases that endanger public’s health. The US Centers for Disease Control and Prevention predicts that 7% of elderly people will eventually develop to ESRD, requiring dialysis or kidney transplantation to survive [2].

Autophagy is a highly conserved lysosomal protein degradation pathway carrying out degradation of cytoplasmic components including protein aggregates, damaged organelles, and even the invading pathogens [3]. In almost all the eukaryotic cells, there are exist basal levels autophagy, which is responsible for maintaining cellular homeostasis by degrading long-lived proteins and damaged organelles [4, 5]. Additionally, autophagy can also be induced by metabolic, genotoxic, or hypoxic stress cues and function as an adaptive mechanism, which is essential for cell survival [4, 5]. Both the two types of autophagy are involved in many human diseases, including neurodegenerative diseases, cancer, inflammatory and autoimmune diseases, and renal fibrosis [5-12].

The activation of autophagy plays a protective effect on renal cells under stress condition [13, 14], and the deficiency of autophagy will increase the sensitivity of the kidney to the damage, leading to impaired renal function, accumulation of damaged mitochondria, premature of kidney and more severe of renal fibrosis [15-18]. However, other studies have found that the continuously activation of autophagy was harmful for kidney after severe injuries, leading to renal cell senescence and promoting renal fibrosis through secreting of profibrotic cytokins[19, 20]. Therefore, the specific role of autophagy in renal fibrosis remains unknown. With the use of conditional autophagy-related genes (ATGs), such as Atg5 or Atg7, knockout mice, accompanied by the construction of animal disease models and the improvement of methods for monitoring autophagy, future research on autophagy in renal fibrosis will be further elucidated. Here, we will discuss the recent advances of the autophagy pathway and highlight its role in renal fibrosis. Moreover, we will also discuss the problems existing in this filed and the possibility of autophagy as a promising therapeutic target for renal fibrosis.

## 2.The autophagy pathway

Autophagy is a process in which eukaryotic cells degrade their own cytoplasmic proteins and damaged organelles or invading pathogens through lysosomes under the regulation of ATGs, thereby maintaining cell homeostasis and cell integrity [21, 22].

**Figure 1.**
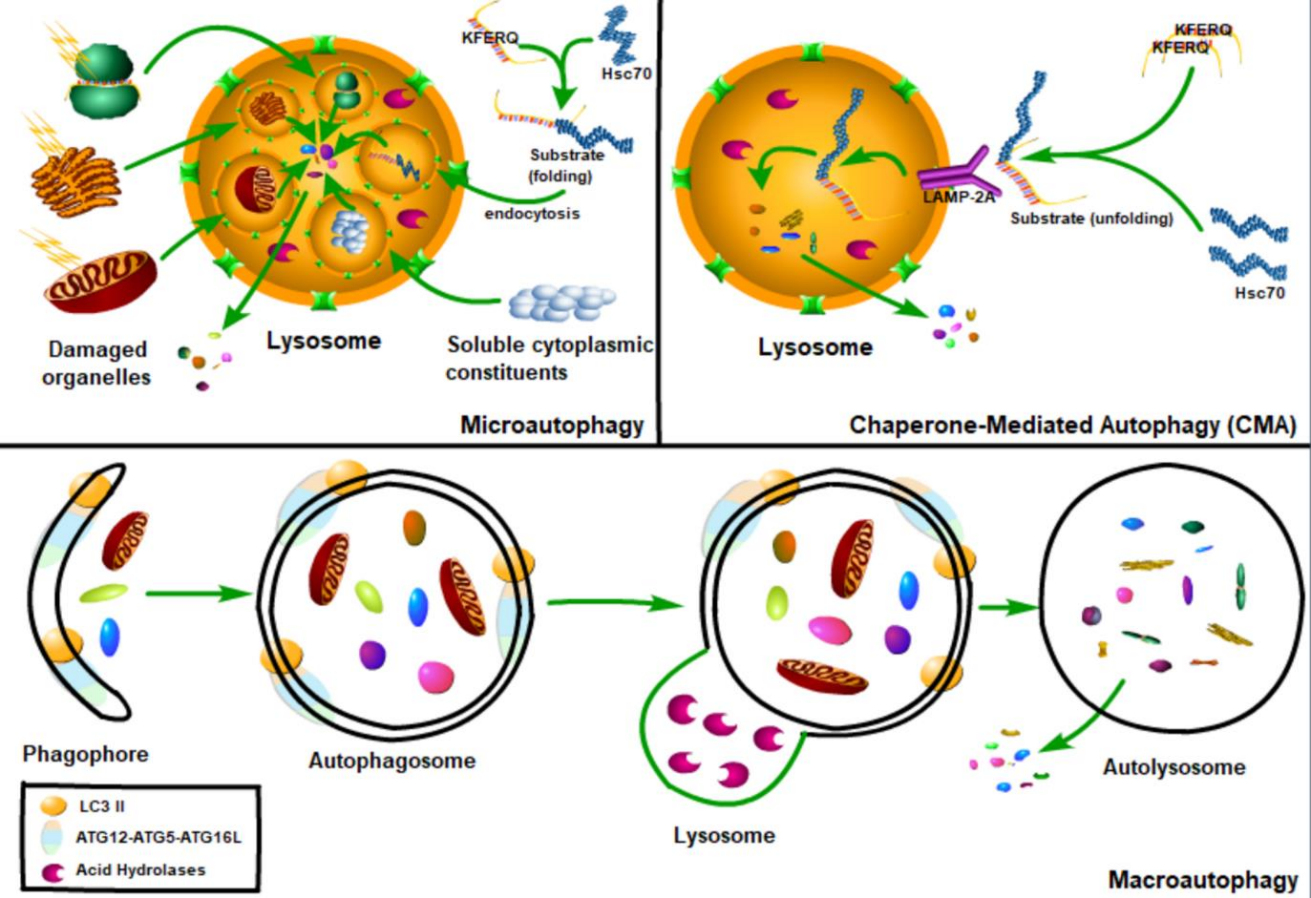
The classification of autophagy. Autophagy can be divided into three types: macroautophagy, microautophagy and chaperone-mediated autophagy. Macroautophagy refers to the phagocytosis of large cytoplasmic materials by autophagosomes, and then fusion with lysosomes to degrade substrates. Microautophagy refers to the lysosome itself engulfing small components of the cytoplasm by invading the lysosome membrane. And the chaperone-mediated autophagy, the chaperone protein Hsc70 (heat shock cognate 70) and the accessory chaperone protein specifically recognize the cytoplasmic protein containing KFERQ-like pentapeptide, and then pass through the lysosomal-associated membrane glycoprotein 2A (LAMP2A) interaction, the unfolded protein is transported into the lysosome cavity through the multimeric translocation complex.

### 2.1 The classification of autophagy

According to the functions and the mode of cargo delivery to the lysosome, autophagy can be divided into three types: macroautophagy, microautophagy and chaperone-mediated autophagy (Fig. 1). Macroautophagy refers to the phagocytosis of large cytoplasmic materials by autophagosomes, and then fusion with lysosomes to degrade substrates. Microautophagy refers to the lysosome itself engulfing small components of the cytoplasm by invaginating the lysosome membrane [23]. In chaperone-mediated autophagy, the chaperone protein heat shock cognate 70 (Hsc70) and the accessory chaperone protein specifically recognize the cytoplasmic protein containing KFERQ-like pentapeptide, and then interact with the lysosomal-associated membrane glycoprotein 2A (LAMP2A), by which the unfolded protein is finally transported into the lysosome cavity through the multimeric translocation complex [22] (Fig. 1). Compared with microautophagy and chaperone-mediated autophagy, macroautophagy is the most widely studied autophagy, therefore the macroautophagy is referred to simply as autophagy hereafter [23].

**Figure 2.**
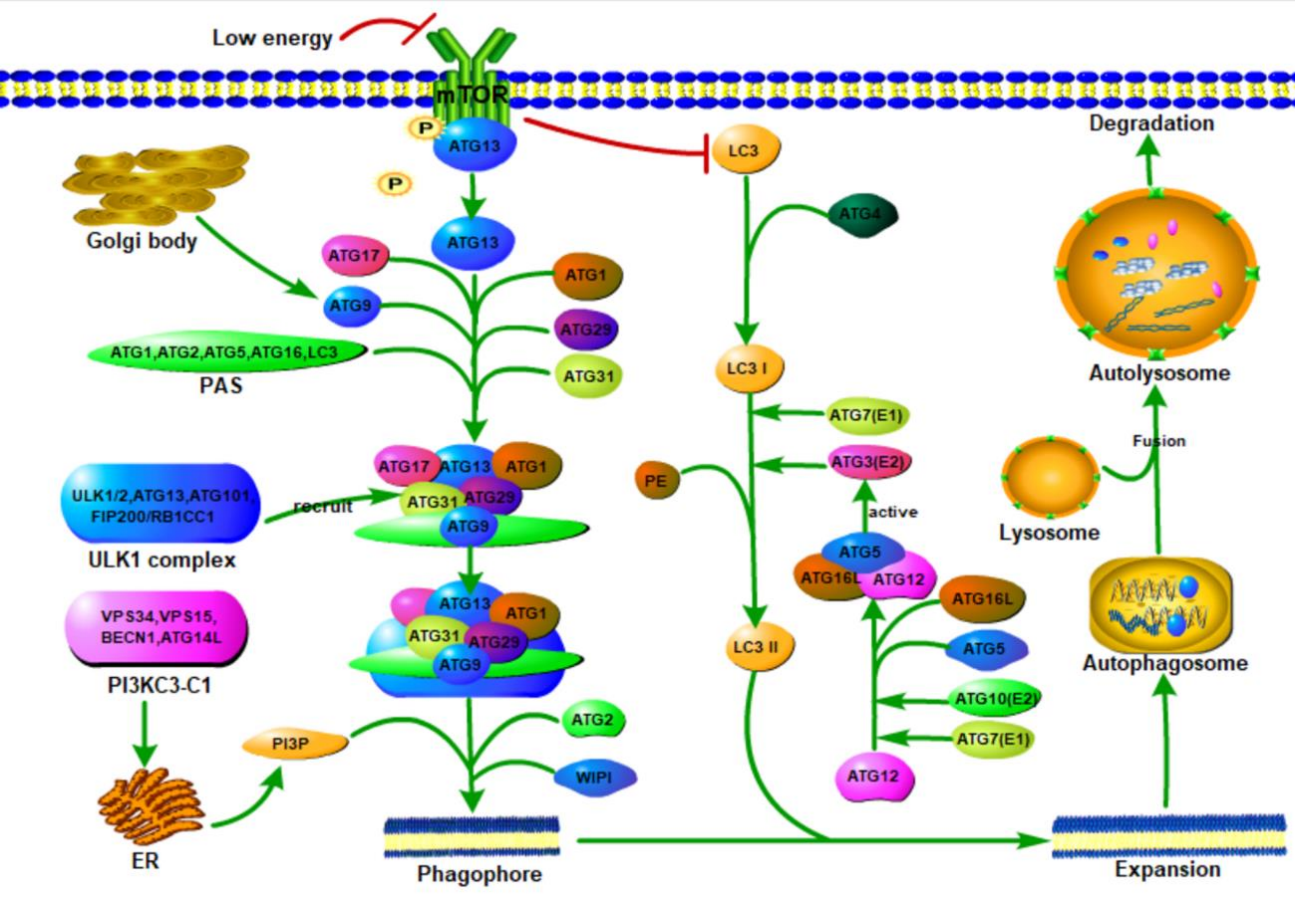
The formation of autophagy. Autophagy is a multi-step process involving initiation, nucleation, expansion, fusion and degradation. When starvation or treatment with rapamycin, ATG13 dephosphorylates and binds to ATG17 in a mTOR protein-dependent manner and activates ATG1 to induce autophagy. Then ATG1 and ATG13 interact with ATG17, ATG29, and ATG31 complexes to form a PAS scaffold complex, which is a prerequisite step for the assembly of ATG protein downstream of PAS. The ULK/ATG1 complex is recruited into the membrane structure independently of PI3P and its downstream ATG protein, and then it is stabilized in the membrane structure by PI3P. ATG9 vesicles, deriving from the Golgi apparatus, can provide lipids required for downstream protein assembly of PAS, recruit ULK/ATG1 complexes, initiate autophagy and serve as a source of autophagosome membranes. Class III PI3K complex I (PI3KC3-C1) is necessary for the nucleation of autophagosomes and is composed of Vps34/VPS34, Vps15/p150, Vps30/BECN1 and Atg14/ATG14L. During autophagy induction, PI3KC3-C1, which produces PI3P on PAS, is recruited into PAS. PI3P transmits the received signal to the downstream ATG proteins through ATG18/WIPI protein. Two ubiquitin-like binding systems, the Atg8/LC3 system and the ATG12-ATG5-ATG16L system regulate the expansion and completion of autophagosomes. when starvation, mTOR protein is inactivated and starts LC3II transcription. LC3 is transformed into LC3I under the processing of ATG4, and then binds to PE under the catalysis of E1-like enzyme ATG7 and E2-like enzyme ATG3 (activated by ATG12-ATG5-ATG16L) and participates in the expansion and completion of autophagosome. After the phagocytic vesicles are expanded and expanded to form autophagosomes, only after the outer membrane of the autophagosomes fuse with the lysosome and complete the degradation of the contents by lysosomal hydrolases. The degraded substrate is eventually released into the cytoplasm for reuse. E1, ubiquitin-activating enzyme; E2, ubiquitin-conjugating enzyme; E3, ubiquitin ligase; ER, endoplasmic reticulum; PE, phosphatidylethanolamine.

Autophagy can also be divided into basal autophagy under physiological conditions and induced autophagy under stress conditions. Basal autophagy is important for maintaining normal cellular homeostasis by removing potentially dysfunctional organelles and longevity proteins. Whereas environmental and intracellular stress induced autophagy is an adaptive response to ensure cell survival and plays essential roles in aging, cancer, neurodegenerative diseases, and infections [5-7, 24].

Additionally, autophagy can also be divided into non-selective autophagy (bulk autophagy) and selective autophagy according to whether the substrate is selectived [25]. Non-selective autophagy degrades cytoplasmic materials non-selectively in response to nutrient starvation, whereas selective autophagy selectively degrades certain cargoes, such as damaged organelles, protein aggregates, or invading pathogens[26-28]. Either non-selective autophagy and selective autophagy or basal autophagy and induced auotphagy should be tightly regulated as dysregulation of these processes involves in many human diseases including renal fibrosis.

### 2.2 The process of autophagy

Autophagy is a tightly regulated and complicated process involving initiation, nucleation, expansion, fusion and degradation. Several different complexes composed by ATG proteins function in coordination with membrane transport components in different steps of autophagosome biogenesis. Multiple ATG proteins control the formation of autophagosomes. So far, more than 40 ATG proteins have been identified in yeast, among which Atg1-10, 12-14, 16, 18 are "core ATG proteins" [29]. Several Atg proteins accumulate to form a special structure called pre-autophagosome structure (PAS). Atg1, Atg2, Atg5, Atg8 and Atg16 are thought to be present in PAS and are directly involved in the formation of autophagosomes [30]. Under the stimulation of starvation, PAS will assemble to form a cup-shaped membrane, the isolation membrane, which will extends and eventually matures into a closed autophagosome [31].

### 2.3 Autophagy inducer and autophagy signaling

The ATG proteins can form six functional groups, namely Atg1 protein kinase complex, class III phos-phatidylinositol 3-kinase (PI3K) complex, Atg9, the Atg18-Atg2 complex and the two ubiquitin-like binding systems (Atg12 binding system and the Atg8 lipidation system) [30, 32]. The complex composed by serine/threonine protein kinase ULK1, ULK2 and other proteins is the major complex for initiating autophagy, while the PI3K complex regulates the nucleation of vesicles and the formation of phagocytic vesicles [33, 34]. Atg9 vesicles, which is derived from the Golgi apparatus, can recruit ULK/Atg1 complexes, initiate autophagy and serve as a source of autophagosome membranes [35-37]. The WIPI-Atg2 complex transmits the received signal to the downstream ATG proteins by binding to PI3P [37, 38]. Finally, the two ubiquitin-like binding systems, the ATG12-ATG5-ATG16L system and the microtubule-associated protein 1 light chain 3 (MAP1LC3; also known as LC3) system regulate the expansion and completion of autophagosomes [39] (Fig. 2).

### 2.4 The regulation of autophagy

**Figure 3.**
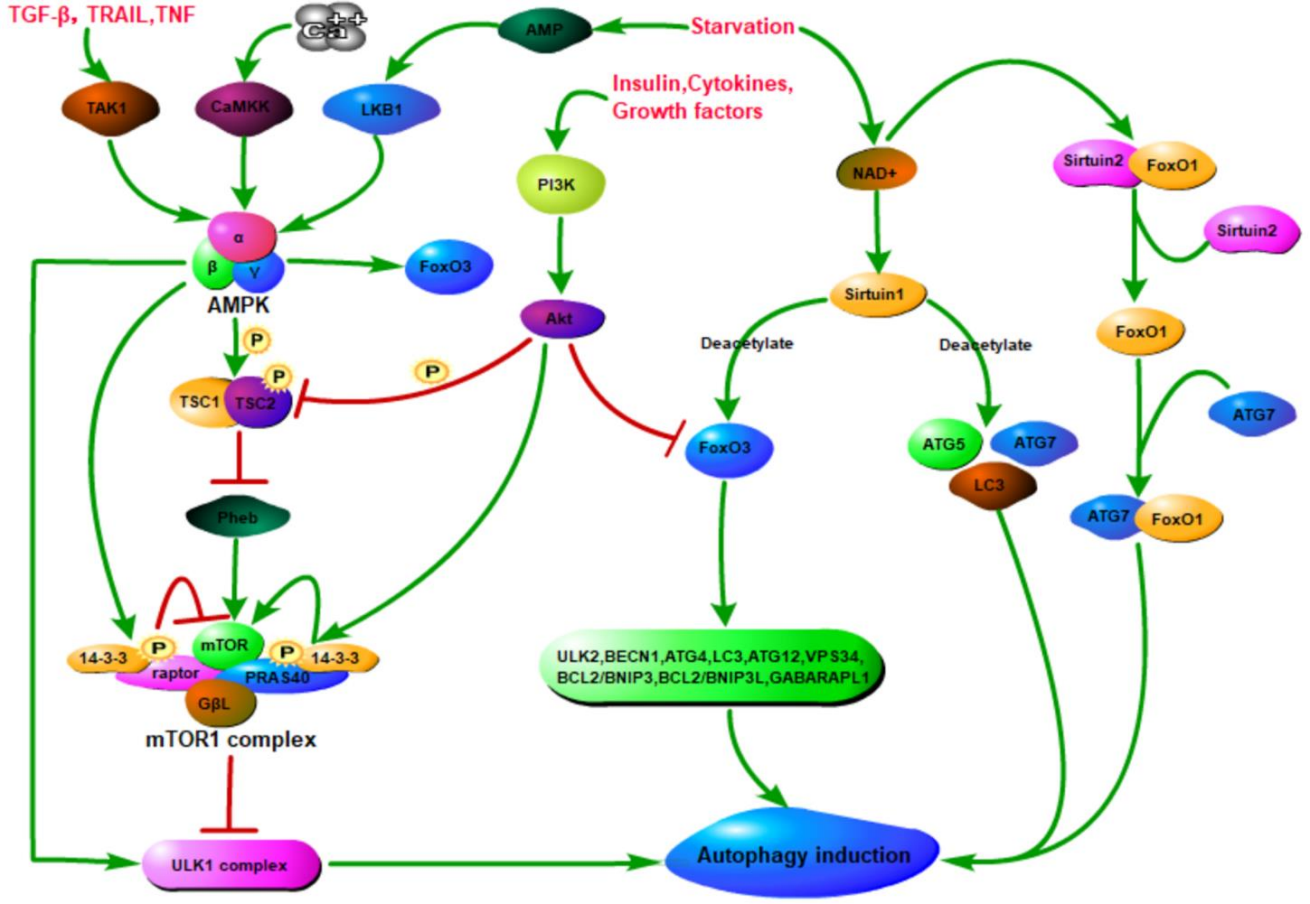
Nutrient dependent autophagy regulation. Nutrition-dependent autophagy regulation is mainly related to mammalian rapamycin target complex 1 (mTORC1), adenosine monophosphate-activated protein kinase (AMPK), and oxidized nicotinamide adenine dinucleotide-dependent histone deacetylase (Sirtuin1). mTOR is the major negative regulator of autophagy. mTOR signaling pathway was activated in nutrient abundance, while AMPK signaling pathway and Sirtuin1 signaling pathway were activated in nutrient deficiency. AMPK is phosphorylated and activated by several upstream kinases, including LKB1, calcium/calmodulin-dependent protein kinase (CAMKK) and mitogen-activated protein kinase 7 (TAK1). Once activated, on the one hand, AMPK can directly phosphorylate serine/threonine protein kinase ULK1 to promote autophagy and/or directly phosphorylate raptor (a scaffold protein used to recruit mTOR substrates) to mediate the binding of 14-3-3 (a cytosolic anchor protein) to inactivate mTOR, thereby indirectly activate autophagy. On the other hand, AMPK can phosphorylate TSC2 in the TSC1/TSC2 complex, disrupt the interaction between TSC1 and TSC2, thereby inhibiting mTORC1 and indirectly activating autophagy. Rheb is located downstream of the TSC1/TSC2 complex and upstream of mTOR and acts as an exciter of mTOR and is inhibited by the TSC1/TSC2 complex. However, in the PI3K/Akt signaling pathway activated by growth factors or cytokines, Akt acts to activate mTOR by inducing the phosphorylation of PRAS40 and mediating its binding to 14-3-3. Moreover, Akt can also inactive TSC2 or inhibit FoxO3 (a transcription factor that can positively regulate autophagy) to suppress autophagy. When starvation, Sirtuin1 and Sirtuin2 are activated due to increased NAD+. Sirtuin 1 can deacetylate forkhead box protein O1 (FoxO1) to promote autophagic flux and/or directly deacetylate several essential autophagy proteins such as ATG5, ATG7 and microtubule-associated protein 1 light chain 3 (LC3) to induce autophagy. In addition, FoxO1 is acetylated after separation from Sirtuin2, and the acetylated FoxO1 promotes autophagy by enhancing its interaction with ATG7.

Autophagy is generally induced by cellular stress, such as hypoxia, reactive oxygen species (ROS), endoplasmic reticulum (ER) stress, DNA damage, nutrient or growth factor deprivation, protein aggregates, damaged organelles, or invading pathogens and immune signalling [40]. Additionally, autophagy is mainly regulated by the three major nutrient-sensing pathways, the mammalian target of rapamycin complex 1 (mTORC1) [41, 42] pathway, the adenosine monophosphate-activated protein kinase (AMPK) pathway [43, 44], and the oxidized nicotinamide adenine dinucleotide-dependent histone deacetylase sirtuin 1(SIRT1)[45] (Fig. 3).

Mammalian target of rapamycin (mTOR) is a serine/threonine protein kinase whose activity is related to redox state and nutrient levels [46]. mTORC1 is considered to be a nutrient and insulin regulating complex, which is consisted by mTOR, raptor (a scaffold protein used to recruit mTOR substrates) [47] and GβL (G-protein β-subunit-like protein; a regulator of mTOR kinase activity) [48]. mTORC2 is involved in cytoskeleton regulation [49] and Akt phosphorylation, and is formed by the combination of mTOR, rictor and GβL [50]. Under the nutrient-rich conditions, GβL constitutively interacts with the mTOR kinase domain independently of raptor, and activating the mTOR kinase [48]. In addition, mTORC1 could interact with the ULK1-Atg13-FIP200 complex and inhibits autophagy induction through the phosphorylation of ULK1 and ATG13 [41, 51] (Fig. 3).

In the low-energy state, cellular autophagy is activated mainly by AMPK signaling pathway and Sirtuin 1 signaling pathway. AMPK is a heterotrimeric complex composed by α, β and γ subunits. The upstream kinases LKB1 and CaMKK could activate AMPK by phosphorylation of its 172-threonine residue. Moreover, the activation of AMPK by LKB1 and CaMKK depends on the AMP/ATP ratio and intracellular calcium ion concentration, respectively [52, 53]. In addition, AMPK can also be activated by TAK1, independent of LKB1 and CaMKK [54]. Upon starvation, the level of adenosine monophosphate in the cell is increasing, accompanied by the increasing of AMP/ATP ratio [55], causing the activation of AMPK, which could activate autophagy through directly activating ULK1 through phosphorylation of Ser 317 and Ser 777 [42] (Fig. 3).

In addition, the activated AMPK can also phosphorylate TSC2 in the TSC1/TSC2 complex, and disrupt the interaction between TSC1 and TSC2, thereby inhibits mTORC1 and indirectly activates autophagy [56, 57]. Moreover, TSC2 has a very high and selective GAP activity to inhibit the function of Rheb, which could phosphorylate mTOR and plays an important role in regulating the state of S6K and 4EBP1 in response to nutrition and cellular energy [58]. Additionally, AMPK can also directly phosphorylate raptor to mediate the binding of 14-3-3 (a cytosolic anchor protein) to inactivate mTOR[56]. However, in the growth factors activated PI3K/Akt signaling pathway, Akt activates mTOR by promoting the phosphorylation of PRAS40 and mediates its binding to 14-3-3[59, 60] (Fig. 3).

Sirtuins, a family of NAD^+^-dependent histone and nonhistone deacetylases, play a fundamental role in sensing and regulating the response of cells to external pressures (such as nutrient availability)[61]. The activation of autophagy associated with Sirtuins is mainly caused by starvation. The induction of autophagy by starvation (but not by rapamycin or ER stress) requires SIRT1 [62]. Seven Sirtuins have been found in mammals. Among them, SIRT1, SIRT6, and SIRT7 are all located in the nucleus; SIRT3, SIRT4, and SIRT5 are mitochondrial sirtuins [63]; SIRT2 is a cytoplasmic sirtuin that is located in the nucleus during the G2/M phase [64].

SIRT1 could deacetylate autophagy proteins, such as ATG5, ATG7 and ATG8, in a nicotinamide adenine dinucleotide-dependent manner, which is essential for the activation of autophagy [45]. In addition, SIRT1 can also indirectly regulate autophagy by deacetylating FoxO (forkhead box O) transcription factors [65, 66], which play a multifaceted role in the regulation of autophagy [67-70]. As the typical function of transcription factors, nuclear FoxOs can induce autophagy by binding to the promoter regions and promoting the expression of autophagy genes [70-73]. Additionally, cytosolic FoxOs can regulate autophagy by interacting directly with autophagy proteins, such as Atg 7, which is independently of transactivation [74-77]. Moreover, FoxOs can also regulate autophagy through epigenetic mechanisms, such as histone modifications and microRNAs [78-81]. Finally, FoxO proteins turnover is controlled directly or indirectly by autophagy [82, 83]. SIRT1 could deacetylate and activate FoxO3, which could upregulate multiple autophagy-related genes, such as ULK2, BECN1, ATG4B, LC3, VPS34, ATG12, BCL2/BNIP3, BCL2/ BNIP3L and GABARAPL1 [40, 84]. Studies have also shown that SIRT1(-/-) mice were partly similar to ATG5(-/-) mice, manifesting as destruction of energy homeostasis, accumulation of damaged organelles and early perinatal mortality [45]. In addition, serum withdrawal could lead to the dissociation of SIRT2 and FoxO1, causing the acetylated FoxO1 increasing, which facilitates its interaction with ATG7 in the cytoplasm and stimulates autophagy [75]. Therefore, Sirtuins can regulate autophagy by directly and indirectly acting on related components that regulate autophagy (Fig. 3).

Additionally, oxidative stress, redox stress, hypoxia, ER stress, growth factor deficiency, exposure to double-stranded RNA viruses, ultraviolet exposure, heme deficiency, immune signalscan and mitochondrial damage could effectively induce autophagy [40, 85]. ER is mainly involved in the synthesis and maturation of proteins (including the correct folding of proteins), among which the unfolded protein response (UPR) is the main factor that activates autophagy under ER stress [86, 87]. Upon ER stress, ER membrane-related proteins, such as PERK, PKR, ATF6, and IRE1, participate in the activation process of autophagy. Among them, PERK induces the activation of autophagy through positive regulation of ATF4, CHOP, NF-κB, and eIF-2κ; PKR induces autophagy by positive regulation of JNK1, eIF-2α, and IKκ; IRE1 activates autophagy through JNK1 and inhibits autophagy by XBP1; whereas, ATF6 could directly activates autophagy [40].

Hypoxia induces autophagy by promoting the up-regulation of HIF-α, DJ-1, PDGF/PDGFR, and PERK[88-91]. The increased levels of ROS could initiate autophagy by activating PERK, ATG4 protease or JNK1. Additionally, ROS can also activate downstream protein p53 and PARP through the DNA damage response-, thereby inducing autophagy [40].

**Figure 4.**
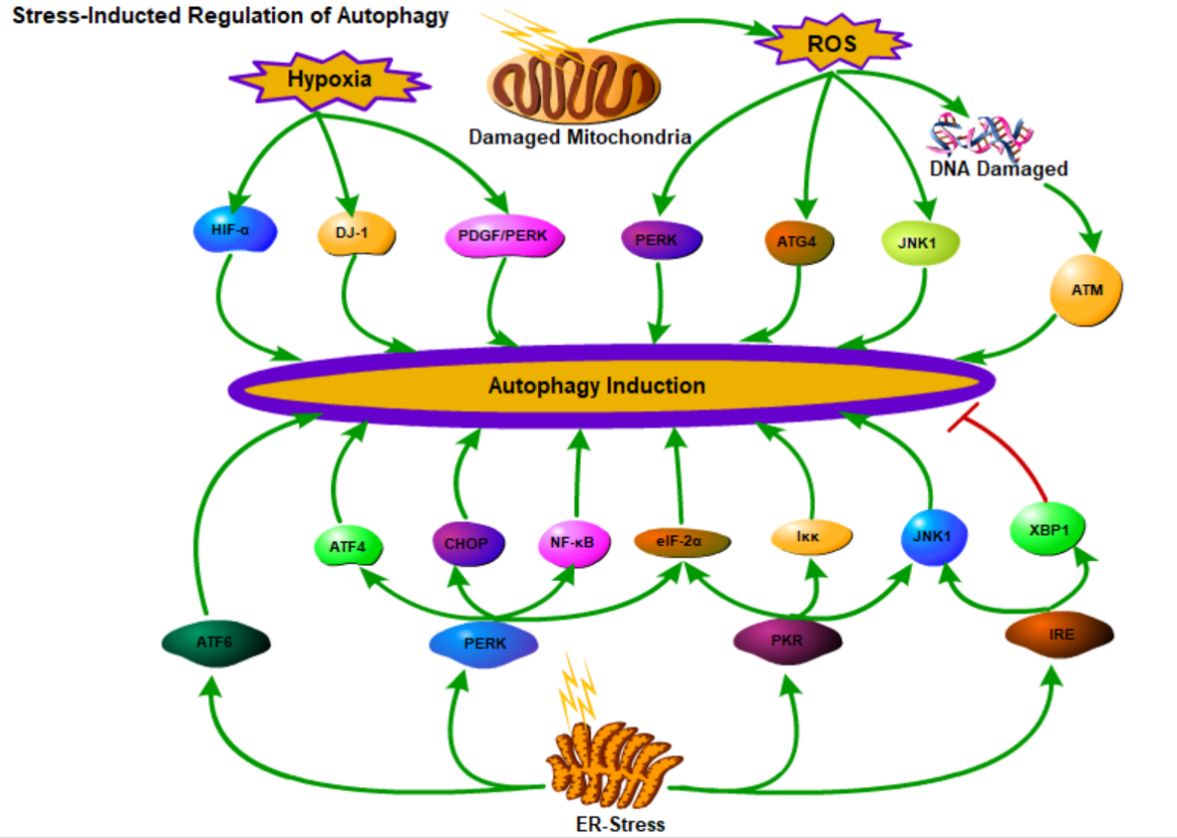
Stress-induced regulation of autophagy. Depicted are the connections between autophagy and ER stress, hypoxia, and the ROS levels.

Cytokines and growth factors could inhibit autophagy independently of the mTOR pathway by activating PI3K/Akt pathway [92]. Whereas, as the downstream effector of the insulin signal, Akt plays dual roles in autophagy, on the one hand it can inhibit autophagy by activating mTOR; on the other hand, it can inhibit autophagy by inhibiting FoxO3 [93] (Fig. 4).

### 2.5 The function of autophagy

Autophagy, an essential catabolic process that degrades cytoplasmic components by the lysosome, is required for a lot of fundamental biological activities, such as the quality control and cellular stress response, especially in postmitotic cells [3].

By facilitating the basal levels turnover of long-lived proteins and organelles such as mitochondria, lysosome, ribosome, ER, and even the nucleus, autophagy plays an important quality control function in the eukaryotic cells [94-97]. This homeostatic function is involved in the pathogenesis of a wide variety of neurodegenerative diseases and age-related diseases [29, 98-101].

Moreover, autophagy is also an important cellular stress response induced by a variety of stresses including nutrient deprivation, growth factor withdrawal, oxidative stress, or infection [40]. In response of these stresses, autophagy is activated so as to maintain cellular biosynthetic capacity and ATP levels by providing amino acids for protein synthesis and substrates for the tricarboxylic acid (TCA) cycle [102].

Therefore, the major role of autophagy is to serve as a quality control system by clearing misfolded proteins and other cellular components under normal physiological conditions, and to provide metabolic precursors for cellular survival in response to various stresses.

## 3. The role of autophagy in liver fibrosis and lung fibrosis

The role of autophagy in liver fibrosis is complex and depends on the cell type in which it is activated [103, 104]. Autophagy in hepatocytes, endothelial cells and macrophages is protective, which can indirectly protect liver from fibrosis [105-107]. Specifically, autophagy inhibits liver fibrosis through alleviating hepatocyte injury, keeping endothelial cell homeostasis, and downregulating the expression of inflammatory cytokines by macrophages and endothelial cells [103, 104]. However, the autophagy of hepatic stellate cells (HSCs) is detrimental, which contributes to liver fibrosis by facilitating lipid droplets brake-up in quiescent HSCs, therefore promoting HSC activation [108, 109].

Idiopathic pulmonary fibrosis (IPF) is a chronic, progressive, and fibrotic lung disease that occured primarily in older adults and often with unknown cause [110-112]. During the process of IPF, the activity of autophagy is decreased, manifesting as concomitant accumulation of p62 and ubiquitinated protein [113]. In vitro studies showed that autophagy inhibition was able to accelerate senescence of epithelial cell and promote myofibroblast differentiation, both of which contributed to lung fibrosis [113]. Compared with control mice, Atg4b-deficient mice exhibited augmented apoptosis of alveolar and bronchiolar epithelial cells and more severe fibrosis manifesting as increased collagen accumulation and deregulated ECM-related gene expression[114]. Deficiency of autophagy in myeloid cells by deletion of Atg5 or Atg7 resulted in more severe lung inflammation and pulmonary fibrosis [115]. These results indicate that autophagy plays an important role in lung inflammation and fibrosis through regulating cellular senescence, differentiation and apoptosis.

## 4. Autophagy in renal fibrosis

Renal fibrosis is the common process of almost all the CKD progressing to ESRD, independent of the underlying etiology [116]. Despite a mass of promising experimental data, currently available therapeutic strategies only mitigate or delay CKD progression but can not reverse renal fibrosis. Besides, the novel antifibrotic strategies could not translate from bench to bedside due to the complexity of the renal fibrosis. Almost all the kidney resident cells, including tubular epithelial cells, podocytes, endothelial cells and mesangial cells, were involved in the pathogenesis of renal fibrosis, indicating the extremely complexity of this process [117, 118].

As an important stress-responsive system, autophagy has been proved to be involved in the pathogenesis of various kidney diseases, including renal fibrosis[84, 119]. Although autophagy is not indispensable in kidney development [15, 120], it plays essential roles in adult kidney resident cells and tightly involved in the progression of renal fibrosis [84, 121] (Table 1).

**Table 1:** Autophagy of different types of kidney cell and renal fibrosis.

Animal Models	Kidney Cell	Renal Disease Model	Renal Function	Renal Phenotype	Autophagy Activity	Fibrosis	Ref.
Conditional deletion of ATG5 in proximal tubular S3 segments	RTECs	30 days after ischemia/reperfusion	Improve	Decreased tubular senescence	Decreased	Alleviation of interstitial fibrosis	[147]
Conditional deletion of ATG5 in proximal tubular S3 segments	RTECs	2 hours after ischemia/reperfusion	-	more cell death as well as tubular damage and inflammation	Decreased	-	[147]
Kidney proximal tubule-specific knockout of ATG7	RTECs	Unilateral ureteral obstruction (UUO)	-	Decreased proliferation and activation of myofibroblasts, decreased ECM components accumulation and tubular atrophy, cell apoptosis, nephron loss, interstitial macrophage infiltration and the expression of a profibrotic factor FGF2 were all inhibited	Decreased	Alleviated	[148]
PTEC-specific deletion of ATG5	RTECs	UUO model	-	Robust ECM deposition and increasing number of RTECs arrested at the cell cycle G2/M phase	Decreased	Remarkably interstitial fibrosis	[132, 133, 150]
Specific deletion of Atg5	Podocytes	Aged	Decline	Accumulation of ubiquitinated proteins, ER stress, podocytes loss, massive proteinuria, more vulnerable to glomerular disease	Decreased	Finally resulting in glomerulosclerosis and renal fibrosis	[120]
Specifically deletion of Atg5 in podocytes	Podocytes	Diabetic nephropathy (DN) model	Decline	Accelerated diabetes-induced podocytopathy, massive proteinuria	Decreased	Glomerulosclerosis	[161]
Specifically deletion of Atg5	Podocytes	high-fat diet (HFD) -induced diabetic model	Decline	Podocyte loss and podocytes apoptosis, massive proteinuria	Decreased	Glomerulosclerosis	[162]
Specifically deletion of Atg5 in GECs, Atg5fl/fl; Cdh5-Cre	Glomerular endothelial cell (GECs)	10 weeks old	Decline	Mild alterations in the glomerular filtration barrier at baseline, slight dilation of glomerular capillaries, discrete podocyte foot process effacements and loss of glomerular endothelial fenestrations accompanied by endothelial cytoplasmic thickening	Decreased	Glomerulosclerosis	[161]
Specifically deletion of Atg5 in GECs, Atg5fl/fl; Cdh5-Cre	Glomerular endothelial cell (GECs)	Diabetes mellitus (DM)		More severe in dilation of glomerular capillaries, endothelial lesions, glomerular basement membrane (GBM) thickening and podocyte foot process broadening and effacement	Decreased	Glomerulosclerosis	[161]
Atg5fl/fl;Tek-Cre, specifically deletion of Atg5	GECs	-	-	Slightly distended capillary loops accompanied by an accumulation of ROS four weeks after birth, a lobular pattern with thickening of the capillary loops and mesangial matrix expansion eight weeks after birth, and died by twelve weeks of age	Decreased	Glomerulosclerosis	[167]
-	Glomerular mesangial cell (GMC)	TGF-β1	-	Decreased GMC apoptosis and promoting its survival	Increased	Decreased	[169]
-	GMC	AGEs	-	GMC senescence	Decrease	Senescence	[172]
-	GMC	Ang II	-	GMC senescence	Increase	Senescence	[173]

### 4.1 Features of renal fibrosis

As the common final pathway of almost all the CKD, renal fibrosis is characterized by the buildup of scar within the parenchyma, causing cortical interstitial expansion, which is the best manifestation of renal functional decline in various kidney diseases, such as CKD, glomerular diseases, and type I diabetic nephropathy [122-124]. Upon a mild injury, the moderate accumulation of extracellular matrix (ECM) may initially contribute to the tissue repair process and can be subsequently resorbed during the process of renal repair. However, when the injuries are more severe or repeated, it will cause persistent deposition of ECM, ultimately disrupting renal architecture, reducing blood supply, and disturbing kidney function. Fibrosis results in the decreasing of the tissue repair capacity and finally causes kidney failure.

Major features of renal fibrosis include: (1) the infiltration of inflammatory cells, such as macrophage and dendritic cells; (2) the activation and proliferation of myofibroblast that from various sources; (3) the production and persistent accumulation of a large amount of ECM components, such as collagens, fibronectin, laminin, glycoproteins, and proteoglycans; (4) and the renal tubular atrophy and microvascular rarefaction [125, 126]. The process of renal fibrosis can be artificially divided into four stages according to the sequence of these destructive events: the priming stage, the activation stage, the execution stage, and the progression stage [125-127]. It is noteworthy that this type of renal fibrosis division is quite arbitrary, because of the fact that realistic renal fibrosis process is an extremely dynamic process, during which many of these events can occur simultaneously.

### 4.2 Renal tubular epithelial cell autophagy and renal fibrosis

Renal tubular epithelial cells (RTECs) are the main resident renal cells that make up the kidneys and are the most vulnerable to a variety of injuries, such as hypoxia, proteinuria or toxins [128, 129]. It has long been believed that RTECs were the victim of renal fibrosis. However, recent studies have found that RTECs also play an essential role in the process of renal fibrosis [128, 130, 131]. Severe injuries cause a large number of RTECs to undergo apoptosis, and some of the RTECs survive and undergo changes, functioning as fibrogenic cells, driving interstitial inflammation and fibrosis through the secretion of various profibrogenic growth factors, such as TGF-β1 and CTGF [132, 133].

As an important cellular homeostatic mechanism, autophagy plays an essential role in the maintenance of RTECs integrity under physiological condition and pathological conditions, such as acute kidney injury (AKI) caused by ischemia/reperfusion (I/R) [15, 134, 135]. The major function of RTECs is electrolyte reabsorption, which is a process that consumes a large amount of energy [136]. In order to satisfy the energy needs for this reabsorption, RTECs contain large quantities of mitochondria, ER, ribosomes, and lysosomes in cytoplasm. The presence of a number of these organelles plays an essential role in the reabsorption and degradation of albumin and other plasma proteins from the glomerular filtrate [137, 138]. Considering the properties and functions of the RTECs, autophagy probably plays essential roles in RTECs under normal physiological conditions.

By analyzing the GFP-LC3 reporter mouse, Liu et al. have found that RTECs have relatively low levels of autophagy under normal physiological conditions [15]. The time-specific deletion of Atg5 in the RTECs results in increasing of serum creatinine levels and inclusion bodies that p62 and ubiquitin positive, and ultrastructural alterations manifesting as the detection of concentric membrane bodies after 4 to 5 months doxycycline administration [15]. RTECs deficiency of autophagy also accumulated with deformed mitochondria and cytoplasmic inclusions, causing the cellular hypertrophy and eventual degeneration [134]. These results indicating that low basal levels autophagy in RTECs can keep cellular homeostasis by regulating the turnover of cellular proteins and organelles.

Autophagy is induced and function as an adaptive and renal protective mechanism for cell survival in cell stress or pathological conditions, such as AKI--a major kidney disease characterized by a rapid loss of renal function induced by renal ischemia-reperfusion, sepsis, and nephrotoxins [15, 134, 135, 139]. By analyzing the GFP-LC3 reporter mouse, it has been found that I/R injury induced significant increasing of LC3 dots number after 24 hours I/R injury, whereas GFP-LC3 dots were unable to be seen in sham-operated mice, indicating that autophagy probably play an essential role in RTECs stress response [15, 134]. Indeed, RTECs deficiency of autophagy dramatically sensitized the kidneys to ischemic injury or cisplatin-induced AKI, leading to impaired kidney function manifesting as significantly increasing of serum urea nitrogen and creatinine levels, accumulation of damaged mitochondria, p62 and ubiquitin positive cytoplasmic inclusions, and increased tubular cell apoptosis [15, 134, 135]. These results suggest that RTEC autophagy play an essential role in maintaining tubular cell integrity during stress conditions by eliminating damaged mitochondria and avoiding the accumulation of aggregate-prone proteins [139].

However, the role of RTEC autophagy in renal fibrosis remains controversial, although extensive studies have been conducted. The basal autophagic activity in RTECs was higher in the aged mouse kidney than in young mouse kidney, whereas autophagic flux of mouse RTECs in response to metabolic stress was declined with aging [140]. RTEC autophagy deficiency and aged mice (24 months old) appeared a remarkably deterioration in kidney function and fibrosis, accompanied by mitochondrial dysfunction, mitochondrial DNA abnormalities and nuclear DNA damage [140]. These results indicate that age-dependent high basal autophagy in RTECs plays an essential role in counteracting kidney aging and fibrosis by keeping mitochondrial homeostasis. Moreover, the blunted autophagic flux in RTECs in response to metabolic stress may involve in age-related kidney diseases, such as CKD and renal fibrosis.

During aging, the kidney structure is changed, manifested as glomerulosclerosis, tubular atrophy and interstitial fibrosis [141]. Additionally, as a hallmark of aging, the damaged organelles and protein aggregates are also accumulated within cells [142]. Moreover, the activity of the autophagy is decreased with age [143], which may induce the failure removal of protein aggregates and organelles, causing the development of age-related diseases, such as CKD and renal fibrosis. Indeed, the kidney is easy to happen structural changes that related to ageing, such as glomerulosclerosis and interstitial fibrosis. The best evidence for this notion is the increased susceptibility to AKI and high prevalence of CKD in the elderly population[144]. The evidence suggests that autophagy is tightly associated with renal fibrosis during aging.

Autophagy could activate cellular senescence [145], whereas RTECs cellular senescence contributes to renal aging and fibrosis [146]. Baisantry et al. investigated the function of RTEC autophagy in RTECs senescence and renal fibrosis. They found that compared with kidneys from control mice, kidneys from RTEC autophagy-deficient mice (conditional deletion of ATG5 in proximal tubular S3 segments) showed a remarkably decreasing of tubular senescence, alleviation of interstitial fibrosis, and superior renal function after 30 days I/R injury [147]. They also analyzed 2 hours and 3 days mouse kidneys after reperfusion and found that compared with control mouse kidneys, RTEC autophagy-deficient mouse kidneys showed more cell death at 2 hours but less tubular damage and inflammation at day 3 [147]. These results indicated that autophagy promotes the severely damaged RTECs to survival, whereas if these severely damaged RTECs persist, which could lead to maladaptive repair and proinflammatory changes, thereby promoting the progression of renal fibrosis.

In the renal fibrosis mouse model, the persistent activation of autophagy in RTECs was found in the unilateral ureteral obstruction (UUO) mouse kidneys [148]. Moreover, compared with control mouse kidneys, degree of fibrosis in RTEC autophagy-deficient mice (kidney proximal tubule-specific knockout of ATG7) kidneys is alleviated, manifested as the decreasing of proliferation and activation of myofibroblasts, the decreasing of ECM components and tubular atrophy; moverover, cell apoptosis, nephron loss, interstitial macrophage infiltration and the expression of a profibrotic factor FGF2 were all inhibited [148]. These results suggested that persistent activation of autophagy in RTECs facilitates renal fibrosis in UUO mouse model by regulating tubular cell death, interstitial inflammation, and most importantly the secretion of profibrotic factors.

However, Li et al. have found that compared with wild-type mice, RTEC autophagy-deficient mouse (PTEC-specific deletion of ATG5) showed remarkably interstitial fibrosis in a UUO model, manifested as robust ECM deposition and increasing number of RTECs arrested at the cell cycle G2/M phase [149]. These results demonstrated that RTECs autophagy prevents renal fibrosis probably through inhibiting RTECs G2/M phase arrest, as the G2/M phase arrested cell undergoing senescence and secreting various of profibrotic cytokines and promoting renal fibrosis [132, 133, 150].

In summary, RTEC autophagy plays protective roles in the maintenance of RTECs integrity under physiological, AKI or ageing conditions. But its role in renal fibrosis is still controversial. RTEC autophagy affects renal fibrosis probably through its effect on cellular senescence, a terminal arrest of proliferation triggered by cellular stresses such as DNA damage, oxidative stress [145]. Senescence not only inhibits the proliferation of damaged cells, but also affects the microenvironment by secreting of profibrotic cytokines (such as TGF-β1 and CTGF), a specialty termed as senescence-associated secretory phenotype (SASP). The RTEC autophagy influences renal fibrosis probably through the SASP of RTECs. Because of autophagy could both activate and inhibit cellular senescence [145], which probably causing the opposite results about the role of RTECs autophagy in renal fibrosis in different mouse model.

### 4.3 Podocyte autophagy and renal fibrosis

Podocytes are the terminally differentiated cells in the glomerulus, which plays an essential role in maintaining the glomerular filtration barrier[151]. These highly differentiated postmitotic epithelial cells have three distinct parts: cell body, major processes, and foot processes [152]. The highly differentiated postmitotic phenotype of podocytes insure the stability of glomerular filtration barrier, but also cause the glomerulus particular vulnerable [153]. Because podocytes are the terminally differentiated cells and unable to proliferation, therefore, podocytes injury and loss is the major cause of massive proteinuria and renal fibrosis [154, 155].

As the major intracellular degradation system, autophagy probably plays an essential role in the maintaining podocyte homeostasis. Indeed, clinical and experimental evidence has shown that the dysfunction of autophagy-lysosome pathway resulted in severe podocyte injury, podocyte loss, massive proteinuria and renal fibrosis [156-158]. Additionally, the basal level of autophagy in podocytes was very high in compared with other kidney resident cells [159, 160], suggesting that autophagy plays an essential role in maintaining podocyte homeostasis and that its dysfunction probably involved in the process of renal fibrosis.

As mentioned above, podocytes have a very high level of constitutive autophagy [120]. Compared with control aged mice, podocyte autophagy-deficient (specific deletion of Atg5 in podocytes) aged mice exhibited glomerulopathy accompanied by an accumulation of ubiquitinated proteins, ER stress, podocytes loss, massive proteinuria, and finally resulting in glomerulosclerosis and renal fibrosis [120]. Autophagy activity increased remarkably in glomeruli from mice induced by proteinuria and in glomeruli from patients with acquired proteinuric diseases. Moreover, compared with control mice, the podocyte autophagy-deficient mice were more vulnerable to glomerular disease. These results suggest that podocytes autophagy (constitutive and induced) is a key protective homeostatic mechanism and plays an essential role in maintaining podocyte integrity, blocking podocyte aging and injury, and in inhibiting renal fibrosis.

Diabetic nephropathy (DN) is a major cause of renal fibrosis and ESRD. The typical process of DN is the appearance of microalbuminuria, the progression to massive proteinuria, and the finally renal fibrosis and renal failure. The massive proteinuria is the leading cause of renal fibrosis and ESRD. Moreover, albuminuria is also a strongly predictive indicator of the CKD including DN. Lenoir et al. found that high glucose promoted podocyte autophagy flux in vitro and in diabetic mice [161]. Compared with control mice, the podocyte autophagy-deficient mice (specifically deletion of Atg5 in podocytes) showed an accelerated diabetes-induced podocytopathy, manifested as massive proteinuria and glomerulosclerosis.

Tagawa et al. also found that podocyte autophagy-deficient mice (specifically deletion of Atg5 in podocytes) showed podocyte loss and massive proteinuria in a high-fat diet (HFD)-induced diabetic model for inducing minimal proteinuria [162]. Moreover, they also showed that podocyte autophagy level was relative insufficient in patients and rats with diabetes and massive proteinuria in compared with those with no or minimal proteinuria. It is worth noting that a mass of damaged lysosomes appeared in the podocytes of diabetic rats with massive proteinuria and HFD-fed, podocyte autophagy-deficient mice. Furthermore, cultured podocytes treated with sera from patients and rats with diabetes and massive proteinuria led to dysfunction of autophagy, causing lysosome dysfunction and podocytes apoptosis [162]. These results indicates that podocyte autophagy plays an important role in maintaining podocytes homeostasis probably by controlling the quality of lysosome under diabetic conditions, and that its impairment results in podocyte loss, massive proteinuria and renal fibrosis.

In summary, podocyte autophagy activity is very high in compared with other kidney resident cells. Moreover, podocytes autophagy (constitutive and induced) plays an essential role in maintaining podocyte integrity, blocking podocyte aging, and inhibiting renal fibrosis.

### 4.4 Glomerular endothelial cell autophagy and renal fibrosis

The kidney contains several kinds of endothelial cells (ECs), such as glomerular endothelial cell (GECs), microvascular ECs within peritubular capillaries and ECs within larger venous and arterial blood vessels [163]. These ECs are exposed to different environments of the kidney and play different transport roles. The GECs, which is highly fenestrated and lined by a fairly thick filamentous glycocalyx, play an essential role in the maintenance of glomerular filtration barrier (GFB) and podocyte structure [163-166].

Lenoir et al. first investigated the function of GEC autophagy in vivo. They found that compared with the 10 weeks old control mice, the same aged GEC autophagy-deficient mice (specifically deletion of Atg5 in GECs, *Atg5^fl/fl^;Cdh5*-Cre) showed mild alterations in the glomerular filtration barrier at baseline, manifesting as slight dilation of glomerular capillaries, discrete podocyte foot process effacements and loss of glomerular endothelial fenestrations accompanied by endothelial cytoplasmic thickening [161]. Moreover, they also analyzed the function of the GEC autophagy in the condition of diabetes mellitus (DM) and found that GEC autophagy-deficient diabetic mice showed more severe in dilation of glomerular capillaries, endothelial lesions, glomerular basement membrane (GBM) thickening and podocyte foot process broadening and effacement in compared with control diabetic mice [161]. These results suggest that GEC autophagy plays a protective role on glomeruli both at baseline and at DM by preserving both endothelial integrity and podocyte ultrastructure to maintain GFB homeostasis.

Matsuda et al. further explored the physiological role of autophagy in GECs, they found that the GECs revealed plentiful autophagic activity, manifested as increasing remarkably in the number of autophagosomes or autolysosomes in GECs after inhibition of autophagy by chloroquine administration [167]. Moreover, they established the other GEC autophagy-deficient mouse line (*Atg5^fl/fl^;Tek*-Cre, specifically deletion of Atg5 in GECs and in hematopoietic cells) and found that these mice exhibited slightly distended capillary loops accompanied by an accumulation of ROS four weeks after birth, a lobular pattern with thickening of the capillary loops and mesangial matrix expansion eight weeks after birth, and died by twelve weeks of age probably due to pancytopenia caused by the defection of their hematopoietic lineages [167].

Furthermore, they performed the bone marrow transplantation experiments, subjecting four weeks old GEC autophagy-deficient mice to irradiation and then followed by bone marrow transplantation from normal control littermates[167]. Twelve months old transplanted mice exhibited mesangiolysis and glomerulosclerosis accompanied by remarkable deterioration of kidney function, manifested as a significant increase in plasma urea nitrogen (UN) and albuminuria. In addition, administration of the ROS scavenger N-acetyl-L-cysteine (NAC) to the GEC autophagy-deficient mice could mitigate the glomerular lesions [167], indicating that GEC autophagy maintained the integrity of glomerular capillaries and inhibited renal fibrosis probably by alleviating oxidative stress.

In summary, GECs exhibit substantial autophagic activity, which was very high in compared with other kidney resident cells. Moreover, GEC autophagy plays an essential role in maintaining GECs integrity, podocyte ultrastructure, GFB homeostasis and integrity of glomerular capillaries. The dysfunction of GEC autophagy could induce renal fibrosis probably due to defective of GFB caused by GECs and podocytes loss.

### 4.5 Glomerular mesangial cell (GMC) autophagy and renal fibrosis

Renal fibrosis is characterized by accumulation of both ECM in renal interstitial and glomerular compartments. As the main cellular constituents of glomerular mesangium, GMCs play an essential role in the progression of glomerular fibrosis and renal fibrosis [168]. As one of the main matrix-producing cells, GMCs secrete mesangial matrix components (such as type IV and type V collagens and fibronectin) associated with the mesangial matrix to form the GBM, the main function of which is to execute the filtration [168]. However, GMCs can be activated by the pathological stimulus, resulting in overproliferation and persistent accumulation of ECM. In addition, the activated GMCs could also secrete various of profibrotic cytokines, such as TGF-β1 and CTGF, all of which are involved in the progression of renal fibrosis.

The function of GMC autophagy in renal fibrosis is poorly understood and most of the studies were in vitro studies. Ding et al. showed that TGF-β1 could induce autophagy, and therefore protected GMCs from apoptosis during serum deprivation [169]. The GMC autophagy probably functions as an adaptive mechanism in response to glomerular injury by inhibiting GMC apoptosis and promoting its survival. Kim et al. have found that GMC autophagy was involved in the intracellular degradation of type I collagen [170]. By negatively regulates and prevents persistent accumulation of ECM, GMC autophagy played an essential role in inhibiting renal fibrosis.

Senescent renal cells increased in various renal diseases, and the increasing of senescent cells promoted the kidney maladaptive repair, causing the progression of renal fibrosis after severe injury [171]. Shi et al. found that AGEs inhibited the activity of autophagy, causing GMC senescence probably through the RAGE/STAT5 pathway [172]. However, Yang et al. showed that autophagy activity was increased in senescent cells treated by Ang II stimulation [173]. Moreover, inhibition of autophagy by 3-methyladenine (3-MA) can reduce GMC senescence that induced by Ang II. In summary, the function of GMC autophagy on renal fibrosis is remain poorly understand, and some in vitro studies have even come to the opposite conclusion. Therefore, it is urgently need for further investigation the role of GMC autophagy in renal fibrosis in vivo by using the specific autophagy-deficient mouse.

## 5. Conclusions and perspectives

The function of autophagy in renal fibrosis has been extensively studied in mouse model, but there is still no consensus and remain controversial probably due to the complexity of renal fibrosis and functional diversity of autophagy. Almost all the kidney resident cells as well as the infiltrated inflammatory cells are involved in the process of renal fibrosis [126]. The function of autophagy in most of these cells and its effect on renal fibrosis remain poorly understand. Autophagy in podocytes and GECs play a protective role in maintaining cell integrity and inhibiting renal fibrosis. However, RTEC autophagy role in renal fibrosis remain controversial, some studies believe that RTEC autophagy can inhibit renal fibrosis whereas the other showed just the opposite. In addition to these studies in knockout mice, there is no strong evidence showed that the autophagy function in other renal resident cells as well as the infiltrated cells play an promoting or inhibiting role in renal fibrosis.

Autophagy of renal resident cells affects renal fibrosis probably through cellular senescence, a conserved program characterized by permanent proliferation arrest and active secretory phenotype [174]. Senescent cells promoting renal fibrosis primarily by secreting a variety of profibrotic factors, such as TGF-β1 and CTGF [171, 175, 176]. Cellular senescence is one of the major causes of aging and responsible for many age-related diseases. The activity of autophagy is reduced in the process of aging, moreover, autophagy also contributes to the extension of the mammalian lifespan and healthspan [143]. Compared with the control mice, the genetically engineered mice with increased the activity of autophagy by Becn1^F121A/F121A^ knock-in or Rubicon knockout have improvement in age-related phenotypes, such as alleviation of cardiac and renal fibrosis, reduced spontaneous tumorigenesis, and extension of the lifespan [177, 178]. In contrast, downregulation the activity of autophagy by deletion of LC3B (LC3^-/-^ mice) or partly deletion of Beclin (Beclin^+/-^ mice) led to increased collagen deposition and increased level of mature profibrotic factor TGF-β in obstructed kidneys [179]. Generally speaking, autophagy could inhibit renal fibrosis by mitigating cell senescence or aging.

The association between senescence and autophagy is complex, and most of the evidences are inferred from the studies in mice or in cultured cells. On the one hand, autophagy could inhibit cellular senescence by eliminating damaged macromolecules or organelles and maintaining cell homeostasis [180-183]. On the other hand, autophagy could promote cellular senescence by formation of the TOR-autophagy spatial coupling compartment (TASCC), which offers a high flux of recycled amino acids and other metabolites, and supports the massive synthesis of the SASP factors [145, 184]. Given the multifaceted functions of autophagy in cellular senescence, it is not entirely surprising that contradictory findings have been reported in renal fibrosis.

Despite substantial data support that autophagy plays an essential role in renal fibrosis, many questions remain unanswered. For example, the role of autophagy in GMCs, fibroblasts cells and macrophages in renal fibrosis remain unknown. The precise mechanisms of autophagy in different types of renal cells in renal fibrosis remain largely unknown. Moreover, the role of microautophagy, chaperone-mediated autophagy and selective autophagy in different types of kidney cells in renal fibrosis is also poorly understood. In addition, most of the current evidences for the function of autophagy in renal fibrosis are come from the mouse models and in vitro cultured cells, whether the results from in vitro cells and animals can be applied to humans remains unknown. In the future, the activation and inhibition of autophagy activity could be a promising therapeutic strategy for renal fibrosis, given its powerful waste disposal function. There is an urgent need to establish a method for monitoring human autophagy activity to evaluate the role of autophagy in human renal fibrosis and the progression of its treatment [185].
